# Neighborhood size-effects shape growing population dynamics in evolutionary public goods games

**DOI:** 10.1038/s42003-019-0299-4

**Published:** 2019-02-05

**Authors:** Gregory J. Kimmel, Philip Gerlee, Joel S. Brown, Philipp M. Altrock

**Affiliations:** 1 Department of Integrated Mathematical Oncology, H. Lee Moffitt Cancer Center and Research Institute, Tampa, FL 33629 USA; 2 Department of Mathematical Sciences, Chalmers University of Technology, Gothenburg, SE-412 96 Sweden; 3 Department of Mathematical Sciences, University of Gothenburg, Gothenburg, SE-412 61 Sweden

## Abstract

An evolutionary game emerges when a subset of individuals incur costs to provide benefits to all individuals. Public goods games (PGG) cover the essence of such dilemmas in which cooperators are prone to exploitation by defectors. We model the population dynamics of a non-linear PGG and consider density-dependence on the global level, while the game occurs within local neighborhoods. At low cooperation, increases in the public good provide increasing returns. At high cooperation, increases provide diminishing returns. This mechanism leads to diverse evolutionarily stable strategies, including monomorphic and polymorphic populations, and neighborhood-size-driven state changes, resulting in hysteresis between equilibria. Stochastic or strategy-dependent variations in neighborhood sizes favor coexistence by destabilizing monomorphic states. We integrate our model with experiments of cancer cell growth and confirm that our framework describes PGG dynamics observed in cellular populations. Our findings advance the understanding of how neighborhood-size effects in PGG shape the dynamics of growing populations.

## Abstract (web summary)

Gregory Kimmel et al. model the population dynamics of public goods games in which interactions occur within local neighborhoods but density dependence is global. They find that neighborhood size drives changes between different evolutionary dynamics and apply their model to dynamics observed in cell populations.

## Introduction

Nature offers many examples where individuals provide public goods that benefit self and others, which can be maintained by population assortment^1^. In banded mongooses, an individual in the foraging line flushes insects for others^2^. Prairie dogs maintain sight lines around their burrows that become available to others in the colony^3^. Predator inspection by guppies^4^, or mobbing of hawks by crows^5^ provide safety to self and others regardless of participation. Via associational refuges, plants defended by spines or toxins may dissuade herbivores from feeding on any plants in a neighborhood^6,7^. Microbes provide public goods by secreting defensive chemicals^8,9^. Yeast synthesize and spill essential nutrients, such as key amino acids into their surroundings^10^. Yeast consuming and synthesizing amino acids for themselves and others were also observed to coevolve in the form of a different social dilemma, the snowdrift game^11^, in which stable coexistence can exist with or without assortment^12^. Cancer cells, too, can engage in public goods games^13–15^. As ecological engineers cancer cells can promote favorable environments^16,17^, suggesting that they evolve cooperation^18,19^, or form mutualistic relationships with other cells^20^. It benefits a cancer cell to recruit blood vessels, signal normal cells and defend against the immune system, which benefit neighbors too. Such interactions can strongly influence the eco-evolutionary dynamics between cooperators (public good producers) and defectors (free-riders).

Benefits that are produced and shared are often only available within a finite neighborhood^21,22^. These finite neighborhoods create a form of assortative interaction: an individual always experiences a slightly higher frequency of its own strategy within a neighborhood than present in the entire population. This tilt is because the individual contributes to the public good if it is a cooperator, and detracts from the collective public good if it is a defector. Furthermore, cooperators may interact more locally than defectors. We are interested in how strategy-specific neighborhood sizes influence the population dynamics and the evolutionarily stable strategy (ESS) of public goods games. In addition, public goods game studies often assume a linear relationship between benefits and the number of cooperators^23,24^. Yet this collective benefit may be nonlinear^25–27^. If one alarm call is sufficient to alert the colony of prairie dogs to a predator, then there are rapid diminishing returns to having several callers. If a collective defense by microbes against a predator, or by cancer cells against the immune system, requires a threshold effect, then there may be increasing returns where two producers more than double the collective benefit.

Here, we considered a nonlinear relationship between number of cooperators and the resulting collective benefit. Using this nonlinear relationship we integrated density-dependent population growth and frequency-dependent local interactions into a single modeling framework. We found that the resulting nonlinear intrinsic growth functions allow a diverse space of evolutionarily stable strategies. In addition to parameter regions in which cooperators can evade the tragedy of the commons, neighborhood-size-driven state switching occurred, which resulted in hysteresis between the monomorphic and polymorphic strategies if the neighborhood size was varied. We also found that stochastic fluctuations in the neighborhood size favored polymorphic strategies. To incorporate empirical evidence, we revisited recent experimental findings of non-linearities in public goods games played by IGF-II producer and non-producer pancreatic cancer cells in vitro^14^, which suggests that our framework can be used to identify critical neighborhood sizes. We also tested the sensitivity of our model by considering its stochastic analog, in which one does not have to chose the neighborhood size exogenously, and we touched on the emerging question of how strategy-specific neighborhood sizes alter the available ESS. Whether higher multi-cellular organisms with complex social structure, yeast, or cancer cells, populations exhibit ecological dynamics (changes in population size), and evolutionary dynamics (changes in relative abundance). The framework we develop here sheds new light on the conditions needed to maintain cooperative traits that provide public good to the environment.

## Results

### Nonlinear growth rate and neighborhood size shape selection

**Fig. 1:**
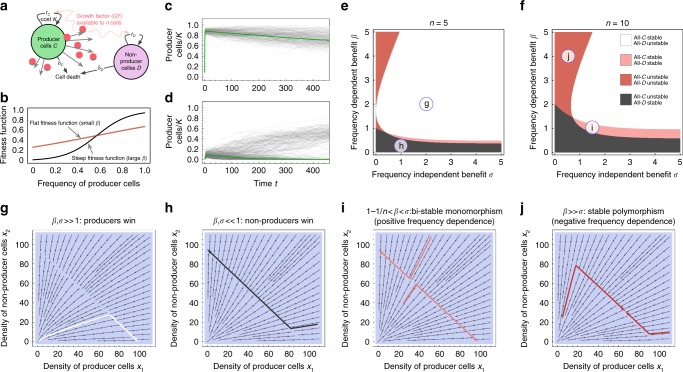
Non-linear growth rate and neighborhood size determine the direction of selection in both stochastic and deterministic public goods population dynamics. **a** Schematic of the eco-evolutionary model, in which cooperators cells (type *C*) provide a public good-like growth factor (GF) at a cost, which benefits both cooperators and defectors (type *D*) within a defined neighborhood of *n* cells, resembling an effective diffusion range of the public good. **b** *β* modulates the frequency-dependent effect, *σ* modulates a frequency-independent (background) effect, and we show examples of nonlinear selection functions that determine the frequency-dependent selection effect on the intrinsic growth rates, (10) with *n* = 8, *σ* = *β*/2, *β* = 2 (red) and *β* = 8 (black). **c**, **d** Stochastic simulations (200 independent trajectories with *n* = 15, *K* = 1000, *α* = 1.0/day, *κ* = 0.5/day, and **c**: *σ* = 2, *β* = 5, leading to coexistence, and **d**: *σ* = 3, *β* = 2, leading to alternative stable states). The green solid lines indicate the mean field dynamics Equations (2) and (4). In **d** we see that stochastic branching is possible, which corresponds to competing stable states–a few stochastic runs made it past the threshold needed to observe long-term coexistence. **e**, **f** Phase diagrams of the co-evolutionary dynamics for three different neighborhood sizes (given in the panels). Smaller neighborhood sizes favor producer cells (white areas). Circles with letters indicate the positions in parameter space used in the following panels. **g**–**j** Stream plots (thin arrows) and example trajectories (thick arrows) of logistic co-evolution (Equations (2) and (4)). The particular parameter settings of the previous panels are marked within their respective phase diagrams. We used: *σ* = 2.0, *β* = 2.0, *n* = 5, leading to dominance of the producers (**g**); *σ* = 1.0, *β* = 0.25, *n* = 5 (**h**), leading to dominance of the free-riders; *σ* = 1.5, *β* = 1.0, *n* = 10 (**i**), leading to unstable polymorphism (bi-stability); *σ* = 0.5, *β* = 4.0, *n* = 10 (**j**), leading to stable polymorphism (coexistence). In all panels we used *α*_*C*,*D*_ = 1.0/day, *δ*_*C*,*D*_ = 0.05/day, *κ* = 0.1/day, and *K* = 100

To investigate the role of nonlinear growth and neighborhood size on the public good game, we considered a population that consists of two sub-populations. Producer cells, *C* (cooperators) produce a public good at a cost and consume it to gain a benefit. Free-rider cells, *D* (defectors), consume available public good, but neither produce nor incur a cost (Fig. 1a). We modeled proliferation and death events as an individual-based stochastic process (see Equation (1), Methods) and in its deterministic limit. The intrinsic growth rates of *C* and *D* are nonlinear functions of the public good shared among a neighborhood of size *n* and the frequency of *C*, which we implemented using two parameters, *β* (frequency-dependent effects) and *σ* (frequency-independent effects). These two parameters modulate the size of the nonlinearities in the growth rates (Fig. 1b) as a function of the frequency (fraction) of *C*. In our model, producers carry a cost *κ* that effectively reduces their intrinsic growth rate irrespective of the environment. Yet, at relatively low neighborhood sizes, producers take advantage of their own public good, as they experience a benefit-to-self. In addition, we considered ecological feed-back in the form of a carrying capacity parameter *K*. Using this model, we observed different ESSs emerge by varying parameters (Fig. 1c, d). Despite a low starting cooperator population, coexistence was still obtained in a few simulations via stochastic branching, which corresponded to passing the threshold needed to enter the long-term coexistence region (Fig. 1d). In the deterministic limit of the stochastic process, the system approaches a set of coupled logistic ordinary differential equations, which describes the dynamics of the two sub-populations, (see Equation (2), Methods).

The neighborhood size *n* also plays a substantial role in determining the number and stability of equilibria, as it shifts the phase boundaries that separate the stable strategies determined by *β* and *σ*. Increasing the neighborhood size favors defectors and decreasing it favors cooperation (Fig. 1e, f). Trajectories of the population dynamics in the deterministic limit unveil the existence of monomorphic and polymorphic strategies, and how these change by modulating the relative values of *β* and *σ* (Fig. 1g–j). These modulations give rise to different hallmark examples of frequency-dependent selection.

In Fig. 1e, f, we give explicit examples of the possible phase diagrams that indicate whether polymorphic equilibria exist and are stable. For fixed neighborhood size, baseline growth rates and cost, these phase diagrams are uniquely determined by the two payoff parameters *β* and *σ*. Increasing the neighborhood size reduces the chances of observing a stable all-*C*. On the other hand, increasing the neighborhood size can increase the parameter range for which we can expect bi-stable evolutionary dynamics (unstable polymorphism). Changes in neighborhood size can critically alter the range of benefit parameters that allow for a stable equilibrium where cooperators and defectors coexist. For example, a stable polymorphic equilibrium can be expected if *β* is an order of magnitude larger than the parameter *σ*, in which case the net growth rate advantage of cooperators is either non-monotonic, or decreases as the abundance of cooperators increases.

### Public goods form determines stability and number of equilibria

If the cost of cooperation is exorbitantly high, the all-*D* state is generally the only ESS. Since defectors do not carry a cost, they always fare better in the absence of any measurable public good effect. The stability of the all-*D* equilibrium is independent of the functional form of the growth rates and depends solely on whether defectors have a higher overall intrinsic growth rate than cooperators. In contrast, the stability of the all-*C* state depends critically on the functional form of the intrinsic growth rates. If *r*_*C*_ > *r*_*D*_ for all frequencies of cooperators in the population, then all-*C* is the ESS. A polymorphic ESS with a mix of *C* and *D* becomes possible when the ratio of respective growth rates is equal to the ratio of death rates *r*_*C*_/*r*_*D*_ = *δ*_*C*_/*δ*_*D*_ for some frequency of cooperators. We label the fraction of cooperators in an equilibrium polymorphic state y^*^, and the total population size in this state Y^*^. When all baseline growth and death rates are the same (*α*_*C*_ = *α*_*D*_ and *δ*_*C*_ = *δ*_*D*_), Y^*^ is largest in the all-*C* state. A full linear stability analysis is presented in Supplementary Methods 4.

Next, let us analyze the number of possible polymorphic equilibria. We show that for the “almost identical” public good functions (*r*_*C*_ = *Ar*_*D*_, for some constant *A* > 0) there can only be one internal equilibrium. The same holds for public good functions that are “always better” in terms of differential returns from the public good, e.g., a cooperator benefits more when another cooperator is added to the neighborhood, $r_C^\prime (y) > r_D^\prime (y)$. Our choice of sigmoidal public good functions Equation (4), can have a maximum of two polymorphic equilibria. For details, see Supplementary Methods 4.2.

### Modulation of neighborhood-size drives state-switching

**Fig. 2:**
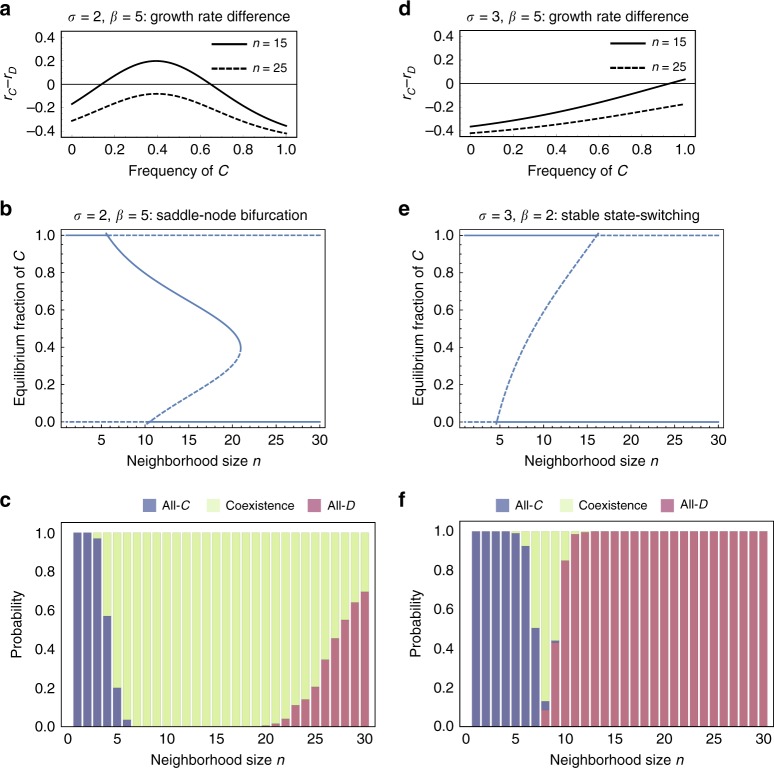
The nonlinear public goods function influences growth rate differences and long-term stable states. **a**–**c** For *β* > *σ*, differences in intrinsic growth rates can have a maximum at a fraction of cooperators between 0 and 1. The number and positions of equilibria then critically depends on neighborhood size, and saddle-node bifurcations are possible. These patterns are also reflected in the individual-based model, revealing large regions of coexistence (shown in **c**). **d**–**f** For *β* < *σ*, differences in intrinsic growth rates tend to be monotonically increasing, and we can observe unstable coexistence and a form of a transcritical bifurcation pattern. The individual based model switches sharply from all-*C* to all-*D* (shown in **f**). We used *α*_*C,D*_  =  1.0, *δ*_*C,D*_  =  0.1, *κ*  =  0.5, and *K*  =  1000. Stochastic simulation results were obtain from 200 independent simulations started from 90 *C* and 10 *D* cells

One internal equilibrium is typically unstable and the other is stable. The stability properties of internal equilibria critically depend on the neighborhood size *n*. This means that saddle-node and transcritical bifurcations^28^ are possible in which the neighborhood size *n* acts as the bifurcation parameter (Fig. 2). The parameter *β* can also control such bifurcations.

The growth rates can be non-monotonic functions of the fraction of *C* in the population. Then, an unstable and a stable polymorphic state can coalesce and annihilate each other at a critical value of neighborhood size. This is known as a saddle-node bifurcation and occurs without affecting the stability of the monomorphic states. Saddle-node bifurcations tend to co-occur with rather sharp transitions between no benefit (due to low numbers of cooperators) to a maximal benefit. Such sharp transitions can be observed for strong frequency-dependent selection (large values of *β*). In Fig. 2a, b we show an example of *β* > *σ* leading to a non-monotonic growth rate differential, which creates a saddle-node bifurcation controlled by the neighborhood size. The stochastic dynamics in this case exhibit a large region of long-term coexistence before large neighborhood sizes favor *D* (Fig. 2c).

In contrast, for *β* < *σ*, we observe monotonically increasing growth rate differences, leading to unstable coexistence. This pattern leads to a transcritical bifurcation (Fig. 2d, e) where the resulting ESSs are alternative stable states of all-*C* or all-*D*. Correspondingly, the stochastic dynamics (Fig. 2f) shows a sharp transition from all-*C* to all-*D* with increasing *n*, with little room for long-term coexistence. In this parameter regime, for *n* = 10, we see that about 10% of the stochastic simulations lead to coexistence, which is also corroborated by the example trajectories shown in Fig. 1d.

### Model integration with in vitro cancer cell growth kinetics

**Fig. 3:**
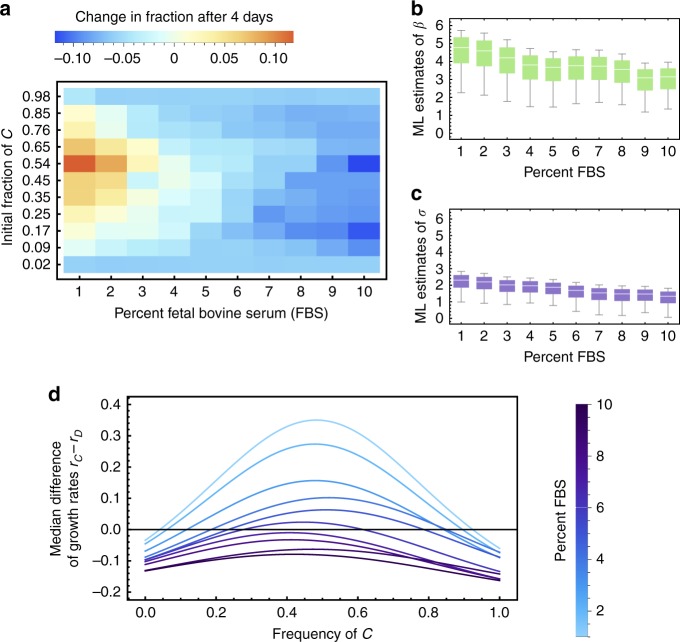
Parameterizing non-linear growth rates using in vitro experiments. **a** Data on the frequency change of *C*, measured over four days by Archetti et al.^14^; using *β*-tumor cell lines derived from the Rip1Tag mouse model^51^ with and without deletion of a gene responsible for production of the growth factor IGF-II, which was identified as the nonlinear public good. The two cell lines were grown in vitro with different concentrations of Fetal Bovine Serum (FBS). **b**, **c** We varied *n* from 4 to 40 cells and fitted a nonlinear model in form of the numerical solution of Supplementary Equation (S18a), to obtain maximum likelihood (ML) estimates of *β* and *σ* for each FBS concentration. **d** Growth rate differences of our re-analysis of Archetti et al.‘s data (with a background growth rate set to 1/day, and using their estimated cost of public good production of *κ* = 0.25*α*, see Fig. 1 in ref. ^14^, and an average neighborhood size *n* = 22). Low concentrations of FBS led to both stable and unstable coexistence, which was lost for higher FBS concentrations

The case *β* > *σ* is of particular interest as it can lead to stable and unstable internal coexistence points due to non-monotonicity in growth rate differences. Public good functions that influence population growth rates in a non-monotonic fashion have been proposed in the context of bacterial, yeast and pancreatic cancer cell monolayers, and were subsequently measured empirically in an in vitro context^14^. However, a concise numerical and statistical interpretation of this data was not included. With our model, we can in part close this gap and recapitulate the intriguing frequency-dependent cancer cell growth patterns measured by Archetti et al.^14^ They established that IGF-II (insulin-like growth factor 2) can act as a nonlinear public good that comes at a specific cost to cooperators (IGF-II producer cells). In the context of freely available nutrients (Fetal Bovine Serum; FBS), the benefit of producing the public good declines with increasing concentration of FBS (Fig. 3a, see also Fig. 3c in reference^14^). To estimate realistic values of *β* and *σ*, we used data from cellular in vitro competition experiments using the population growth and expansion of IGF-II producer cells (cooperators) and non-producer cells (defectors). The actual neighborhood size was not measured or estimated for these experiments. Therefore, in fitting our model to this data, we considered neighborhood size to be independent and identically distributed between *n* = 4 (nearest neighbors in 2D) and *n* = 40 cells. For each value of *n* and for each experimental setting (percent FBS in the medium), we fit our mathematical modeling framework to the cellular growth data (see Supplementary Methods 5), to estimate values for *β* and *σ*. Our estimated values of *β* ranged from 1.5 to 6 (Fig. 3b), and values of *σ* from 0 to 3 (Fig. 3c). For example, in the context of 5% FBS in the background medium, we observed median values of *β* = 3.67 and *σ* = 1.87. In our model, these parameter values would predict an unstable polymorphic equilibrium near y^*^ = 0.235, and a stable polymorphic equilibrium near y^*^ = 0.784 (Fig. 3d). These estimates depend on the distribution of values of *n*, which highlights the need in future studies to devote more effort into determining the distance over which a public good spreads. This statistical analysis shows that our model framework is capable of explaining the evolutionary dynamics of cellular games with nonlinear public goods and confirms that cancer cell lines can exhibit non-monotonic growth rates which favor local coexistence if the background nutrients are sufficiently sparse (*β* > *σ*).

### Type-dependent neighborhood sizes

**Fig. 4:**
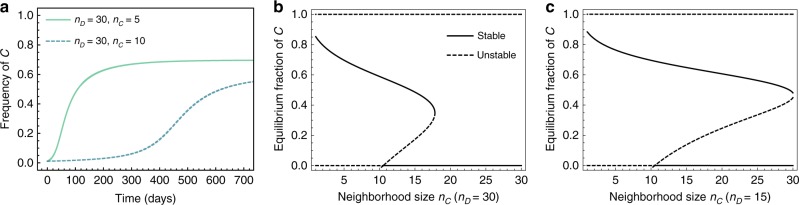
Differences in neighborhood size (public good sensitivity) can destabilize all-*C* but increase the coexistence regime. In all panels: *K* = 1000, *δ*_1_ = *δ*_2_ = 0.1, *γ* = 1.0/day, *σ* = 2.0, *β* = 5.0 and *κ* = 0.5/day. **a** Temporal trajectories of the fraction of cooperators for two different values of *n*_*C*_, where we considered a resident population of defectors (*x*_*D*_(0)/*K* = 1−*δ*/*α*) that is invaded by a small population of cooperators (*x*_*C*_(0) = 10), which move toward coexistence. The time to reach coexistence critically depends on *n*_*D*_ − *n*_*C*_. **b** For fixed *n*_*D*_ = 30 the saddle-node bifurcation pattern of Fig. 3b (where *n*_*D*_ = *n*_*C*_) changes such that the all-*C* state becomes entirely unstable, and we see a shift in the critical value of *n*_*C*_ above which cooperation is lost. **c** Smaller values of, e.g., *n*_*D*_ = 15, act against defectors. Then we predict an increase in range of values of *n*_*C*_ for which cooperators and defectors stably coexist

Cooperators may experience a different neighborhood size, *n*_*C*_, than defectors, *n*_*D*_, for example through aggregation effects^29^, or by altering their local interaction topology^30^. In such cases, defectors could fare better from unconditional interactions with as many cooperators as possible. Thus, with mechanisms that promote increases in neighborhood size at a cost smaller than the cost of cooperation, one could see adaptation toward strategy-dependent neighborhood sizes. Thus, we examined how strategy-specific neighborhood sizes influence coexistence, the ESSs, and the eco-evolutionary dynamics of Equation (2). We assumed that cooperators interact with other individuals within their specific neighborhood of size *n*_*C*_, and that defectors interact within *n*_*D*_. Under these additional assumptions, we examined under which conditions a small population of cooperators can invade a resident population of defectors. The relation *n*_*C*_ < *n*_*D*_ favors coexistence, which can be seen in the different bifurcation patterns in Fig. 4. When both *C* and *D* experience the same neighborhood size, then small sizes favor all-*C* and larger neighborhood sizes favor all-*D*. For a sufficiently small *n*_*C*_, cooperators can invade. For a sufficiently high *n*_*D*_, defectors can invade. Hence $n_C \ll n_D$ reduces the likelihood that all-*D* and/or all-*C* will be an ESS, and increases the likelihood of ESS coexistence. It should be noted that it could be difficult to argue in favor of differences in *n*_*C*_ and *n*_*D*_ in isotropically diffusing growth factors. However, realistic spatial environments, such as a three-dimensional solid tumor with anisotropic collagen patterns could realistically present such a scenario^31^.

### Fluctuations in the neighborhood size influence coexistence

**Fig. 5:**
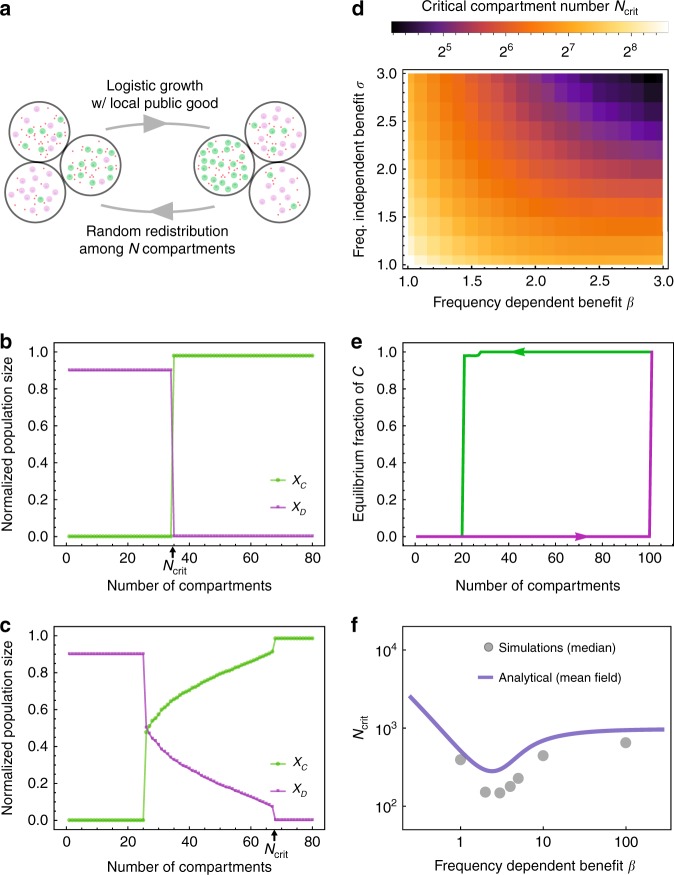
Fluctuations in neighborhood size can stabilize cooperation. **a** Schematic overview of the quasi-spatial model. *N* compartments are filled with producers and free-riders (a compartment can be empty). The system evolves from time *t*_0_ to *t*_0_ + Δ*t* according to the system governed by the Supplementary Equation (S42). We repeatedly “mixed” (multinomial sampling *Y*-times into the *N* compartments) the system followed by selection for a time *T*_*S*_ = 10/*α*. **b** For *β* < *σ* the system suddenly transitions to the all-*C* state with increasing number of compartments (decreasing effective neighborhood size). Parameters: *α* = 1, *β* = 2, *σ* = 3, *κ* = 0.5, *δ*_*C*_ = 0.1, *δ*_*D*_ = 0.1, *K* = 1000, *C*(0) = *D*(0) = 50, *T*_*s*_ = 10. **c** For *β* > *σ* the system transitions to coexistence before equilibrating to the all-*C* state with increasing number of compartments (decreasing effective neighborhood size). Parameters: *α* = 1, *β* = 5, *σ* = 2, *κ* = 0.5, *δ*_*C*_ = 0.1, *δ*_*D*_ = 0.1, *K* = 1000, *C*(0) = *D*(0) = 50, *T*_*s*_ = 10. **d** Calculation of steady state and location of *N*_crit_ for small *β*. The system transitions directly to the producer-only state. Parameters: *α* = 1, *κ* = 0.5, *δ*_*C*_ = 0.1, *δ*_*D*_ = 0.1, *K* = 1000, *C*(0) = *D*(0) = 50, *T*_*s*_ = 10. **e** Hysteresis could be observed when changing *N* without re-initializing the population (parameters are the same as in **b**). **f** Predicted *N*_crit_ using mean-field theory compared to the numerically obtained points. Parameters: *α* = 1, *σ* = 1, *κ* = 0.5, *δ*_*C*_ = 0.1, *δ*_*D*_ = 0.1, *K* = 1000, *C*(0) = *D*(0) = 50, *T*_*s*_ = 10

Here, we are interested in how feedbacks between total population size and neighborhood size influence the ESS of *C* and *D*. To address these questions we devised a compartmentalized version of the eco-evolutionary growth model of Equation (2). Instead of exogenously fixing neighborhood sizes and performing averaging over neighborhood compositions, we let the total population size influence neighborhood sizes by dividing the population into *N* compartments. The total population *Y*, can then be randomly distributed across these compartments, resulting in a multinomial distribution of neighborhood sizes with a mean of *Y*/*N*. The public good is now produced and shared locally among a stochastically sampled number of neighbors. Here, the number of compartments acts as a surrogate for the inverse of an effective neighborhood size, which is an emergent quantity. This stochastic mixing of the population was performed numerically according to a multinomial sampling process (see Supplementary Methods 6). Hence the interaction neighborhood for each strategy type was a stochastic variable determined by the number of compartments and the total population size. A schematic of the compartment-based dynamics is given in Fig. 5a. Of critical importance to the dynamics of this model is the time between the mixing steps (see Supplementary Figures 1–3).

In the compartment approach, we observed that cooperators survived above a threshold number of compartments *N*, and that cooperators would become the ESS above a critical value *N*_crit_. Depending on the values of *β* and *σ*, coexistence of cooperators and defectors was possible. We observed the equivalent of a transcritical bifurcation (switching of stable states), controlled by an increasing number of compartments (Fig. 5b), as well as the equivalent of a saddle-node bifurcation (Fig. 5c) with stable coexistence at intermediate number of compartments. We defined the critical number of compartments such that above *N*_crit_ all-*C* would be stable. Figure 5d shows how *N*_crit_ depends on the nonlinear public goods benefit parameters. Notably, if we did not re-initialize the population when changing the number of compartments, we could observe a strong hysteresis pattern (Fig. 5e) as predicted by the transcritical bifurcation (compare to Fig. 2e).

We used a mean-field approach to estimate *N*_crit_. Instead of using a distribution for the individuals among the *N* compartments, we let each compartment have the expected value, *Y*/*N*. We then determined the number of compartments above which all-*D* becomes unstable and is no longer an ESS. Although this approach misses some important numerical and stochastic fluctuations, it recovers the general trend of the behavior of *N*_crit_ (Fig. 5f), which shows non-monotonic behavior as a function of *β*. For small but increasing *β*, *N*_crit_ decreases monotonically, but then assumes a minimal value at intermediate selection strength. For very strong frequency-dependent selection (large *β*), *N*_crit_ increases asymptotically toward Y^*^. These findings highlight the dynamical feedbacks between population dynamics, neighborhood size, and frequency-dependent effects. These feedbacks also point to how measurements of population densities and interaction distances could determine an effective neighborhood size.

## Discussion

In summary, we modeled both density and frequency-dependent effects in the context of a non-linear public goods game that is effective within a neighborhood. There is a critical role for neighborhood size, which describes the group of individuals that are close enough together to benefit from the public good. Our results demonstrate the following. First, smaller neighborhood sizes favor cooperators and increase the likelihood that all-*C* is an ESS. Second, a linear relationship between the frequency of *C* in the neighborhood and the overall value of the public good permits two global ESSs, which are either globally all-*D* or globally all-*C*, without the possibility of coexistence or alternate stable states. Third, a nonlinear, sigmoidal relationship between the public good and the frequency of *C* allows for multiple stable ESSs. Such nonlinearity was previously measured in cancer cell dynamics^14^, and we made use of their experimental findings to validate our modeling framework. Fourth, under alternate stable states we observe saddle-node and transcritical bifurcations and hysteresis. Last, feedback of population size on neighborhood size and/or strategy-dependent neighborhood sizes can increase the likelihood of coexistence between *C* and *D*. These results from the deterministic approach are corroborated by results obtained from simulations of a corresponding individual-based stochastic model.

Individual-based stochastic models have the benefit that they allow us to estimate extinction probabilities and distributions of the time to extinction. Notably, such systems have often been examined in the fixed population size scenario^32–34^. Recent expansions have focused on emergent phenomena in populations of fluctuating size or coevolutionary feedback^35,36^. These approaches also show that multi-level^37^ or multi-player^38^ population games deserve more attention in the context of fluctuation-driven phenomena. These problems are of interest since population size fluctuations are related to the maintenance of cooperation, extinction delay, or other counterintuitive selection effects^24,39–41^, especially in the context of neighborhood-size dependent access to shared resources.

The number of individuals comprising a neighborhood may be influenced by the total size of the population^42^. In this way, population dynamics can feedback in to the neighborhood size. If neighborhood size increases with population density, then density feedbacks can result in the coexistence of cooperators and defectors. As a consequence, an all-*C* world promotes a higher population size than an all-*D*, as it also allows a higher carrying capacity^24^. With a feedback on neighborhood size, an all-*C* population promotes a higher neighborhood size which, in turn, favors the invasion of defectors. Similarly, an all-*D* population promotes smaller neighborhood sizes that facilitate the ability of cooperators to invade. Density feedbacks have been shown to influence the ESS in games of spite^43^ and producer-scrounger games^44^. However, if the public good is an essential nutrient secreted by a protist or cancer cell, then it may only provide resources for a fixed number of others, regardless of distance from the producer. Hence, there may be little feedback at all. In many cellular interactions, we can expect that a fixed number of nearby neighbors become the beneficiaries of a producer.

Linear public good games do not allow for saddle-node bifurcations, and consequently all-*C* or all-*D* are globally stable ESSs, unless there are density-feedbacks as discussed above. We here show that a nonlinear relationship between the public good and the frequency of producers permits bifurcations and hysteresis between alternative equilibria. We considered a sigmoidal relationship between benefit and cooperator frequency, which can favor coexistence because increasing returns at low fractions of cooperators act in their favor, whereas diminishing returns toward high fractions of cooperators favors defectors. We have observed that there are often maximally two interior equilibria, one will always be unstable and the other representing the stable coexistence of cooperators and defectors. With a different nonlinear relationship, such as a Michaelis–Menten (Hill) function^15^ (see also Supplementary Methods 4.2), there could be up to five interior equilibria.

The multi-player snowdrift game provides another form of public goods game in which players receive a fixed benefit that is not influenced by the frequency of cooperators. Rather, it is the cost that is shared among the cooperators. Hence the cost of cooperating declines with the frequency of cooperators. In our game, it is the opposite. Cooperators bear a fixed cost and it is the benefit that is distributed based on the frequency of cooperators. A similar analysis could be applied to the snowdrift game as we have done here.

Whether it be cancer or other ecological public good games in nature, our results suggest new ways to manipulate the parameters of public good population games. Cancer cells may engage in public goods games in a manner that make the disease more devastating and harder to treat^16,17^. Cells produce signals that promote vasculature, or signal other cells, which can produce vasculature (e.g., vascular endothelial growth factor, VEGF). Then, the recruitment of blood vessels provides a benefit to the producer but also to any cells within some neighborhood of these new vessels. In such systems, however, it is unclear whether there is a significant cost to cooperation. It may be possible to create therapies that incentivize the population game in a manner that shifts the equilibrium towards defectors, for instance by decreasing the benefit of the public good. This approach may have demonstrable value in bicarbonate therapy, which is a means of neutralizing the low pH created by glycolytic cancer cells^45^. Increasing neighborhood size provides an intriguing, yet hitherto unappreciated therapeutic opportunity. Perhaps by increasing the diffusion rate of molecules (including VEGF) within the tumor, the provider cells would experience less of a self-benefit and defectors would perceive a higher benefit. Such manipulations could then achieve control, e.g. of pests or tumors, or promote polymorphism and diversity, e.g. of endangered or valuable species, or maintain cooperative sub-populations.

## Methods

We consider the dynamics of a population that consists of two sub-populations. Producer cells, *C* (cooperators) produce a public good at a cost and consume it to gain a benefit. Free-rider cells, *D* (defectors), consume available public good, but do not produce nor incur a cost. We assume that the total population can grow towards a maximal load, or carrying capacity, which introduces a global density-dependent effect. Proliferation and death events are described by the following process

1a $$C\mathop{\longrightarrow}\limits^{{r_C{\kern 1pt} E_K}}C + C$$

1b $$D\mathop{\longrightarrow}\limits^{{r_D{\kern 1pt} E_K}}D + D$$

1c $$C\mathop{\longrightarrow}\limits^{{\delta _C}}\emptyset$$

1d $$D\mathop{\longrightarrow}\limits^{{\delta _D}}\emptyset$$

where the birth rates *r*_*C*,*D*_ *E*_*K*_ incorporate global density dependence via the factor *E*_*K*_ = 1 − (*C* + *D*)/*K* that is multiplied to the growth rates *r*_*C*,*D*_ (for details, see Supplementary Equation (S2) in Supplementary Methods 1.1). The death rates *δ*_*C*,*D*_ are assumed to be constant. These reactions describe a stochastic individual-based process for which it is possible to derive a mean field dynamical system (see Supplementary Methods 1.2). In the deterministic limit, the densities of cooperators *C* and defectors *D* are given by *x*_*C*_ and *x*_*D*_, and their dynamics are described by the system

2a $$\frac{{{\mathrm d}x_C}}{{{\mathrm d}t}} = r_C\left( {1 - \frac{{x_C + x_D}}{K}} \right){\kern 1pt} x_C - \delta _C\,x_C,$$

2b $$\frac{{{\mathrm d}x_D}}{{{\mathrm d}t}} = r_D\left( {1 - \frac{{x_C + x_D}}{K}} \right){\kern 1pt} x_D - \delta _D\,x_D.$$

We are interested in cases where the intrinsic growth rates depend on the frequency of cooperators *y* = *x*_*C*_/(*x*_*C*_ + *x*_*D*_). There could also be coevolution at the level of *K*^35,46^, which in turn would influence the dynamics differently and is beyond the scope of this manuscript (see comment in the Supplementary Methods 3.2). Here we seek to quantify the impact of available public good on the growth rates, knowing that production can incur a cost on *C* (Fig. 1a).

To calculate how *C* and *D* benefit from public good consider that the public good is a local commodity consumed by all within a neighborhood of *n* cells, for example a growth factor produced by *C*. Thus, we have to account for all possibilities to select up to *n* cooperators. If the fraction of producer cells is *y*, we can obtain the following expected numbers of producer cells of any *C* or *D*

3a $$N_C = \frac{1}{n}\left[ {1 + (n - 1)y} \right],$$

3b $$N_D = \frac{1}{n}(n - 1)y.$$

These values result from hypergeometric sampling *n* − 1 times with a probability *y* to pick *C*, assuming that the population is sufficiently large. The expected values *N*_*C*,*D*_ can then be used to calculate expected growth rates. As we discuss in the Supplementary Methods 1.4 and 2, the use of the hypergeometric distribution to generate a neighborhood and calculate growth rates leads to negligible changes in the dynamics for sufficiently large populations. In the difference between *N*_*C*_ and *N*_*D*_ we see that cooperators might experience an increased benefit, especially if *n* is small, as they are able to benefit from their own public good.

The benefit to each member of a neighborhood may increase nonlinearly with the frequency of cooperators^14,25,47^. One could observe steep increases of fitness at low frequencies of cooperators, followed by a saturation effect in fitness once the fraction of cooperators crosses a threshold. Such a sigmoidal, or s-shaped fitness curve can be described using a parameter *β* that modulates frequency-dependence, and a parameter *σ* that modulates frequency-independence (background). The inflection point where benefit increase is maximal is then given by *σ*/*β*. For *y* < *σ*/*β*, additional cooperators create synergies. For *y* > *σ*/*β*, we see diminishing returns. Including the cost for cooperation, *κ*, we can now write the frequency-dependent intrinsic growth rates as

4a $$r_C = \alpha _C\frac{{1 + {\mathrm{e}}^\sigma }}{{1 + {\mathrm{e}}^{\sigma - \beta N_C}}} - \kappa ,$$

4b $$r_D = \alpha _D\frac{{1 + {\mathrm{e}}^\sigma }}{{1 + {\mathrm{e}}^{\sigma - \beta N_D}.}}$$

For a sketch of these sigmoidal growth rates, see Fig. 1b. Without any frequency-dependent benefit, *β* = 0, we obtain the intrinsic growth rates *r*_*C*_ = *α*_*C*_ − *κ* and *r*_*D*_ = *α*_*D*_. For a very small *β* one can linearize the intrinsic growth rates *r*_*D*_ = *α*_*C*_(1 + *N*_*C*_(*β*)) − *κ*, and *r*_*D*_ = *α*_*D*_(1 + *N*_*D*_(*β*)). Linear relationships between benefits and the amount of the public good have frequently been used recently^24,48–50^. For mathematical convenience we here chosen to implement intrinsic growth rates as a function of the expected number of cooperators. Instead, one can simulate the full stochastic dynamics in relation to the mean field model, or chose to average over group compositions at a different point. These changes are mostly quantitative, impacting only the location of bifurcations in the phase diagrams, the morphology remains the same.

Our mean field approach averages group compositions before calculating the non-linear benefits to the growth rates, which reflects the fully stochastic dynamics rather well (shown in Fig. 1c, d). In addition, one can ask how the complex structure of equilibria of the deterministic system, as modulated by the parameters *β* and *σ* (Fig. 1e–j), is altered by changing the assumptions that led to *N*_*C*,*D*_ and *r*_*C*,*D*_. In Supplementary Figure 4, we show that these changes can be expected to be minimal.

We also explored fluctuations in neighborhood size. Implementing fluctuations in neighborhood size removes undesired effects when calculating expected growth rates: instead of considering *n* as an exogenous parameter, we defined a fixed number *N* of compartments among which all cells are randomly distributed to form neighborhoods. By creating compartments (patches), these local patches become the neighborhoods observed by the cells within, averaging is not applied. In this way, neighborhood sizes vary according to a multinomial distribution with a mean of (*x*_*C*_ + *x*_*D*_)/*N*. To simulate this, we ran a dynamic loop of two steps. First, we evaluated the outcome of a public goods game and logistic growth within each compartment for a fixed amount of time Δ*t*, according to Equation (2) with *n*_*i*_ and *y*_*i*_ in compartment *i*. Second, we modeled neighborhood assembly by pooling individuals from all compartments and re-distributing them randomly among compartments.

We used the approaches described here to examine how the nonlinear growth rates and neighborhood size changes determine the number and stability properties of equilibria (ESSs) of the deterministic system, corroborated by results of the individual-based approach and the compartment approach. All mathematical and computational methods and proofs are detailed further in the Supplementary Information.

### Reporting Summary

Further information on experimental design is available in the Nature Research Reporting Summary linked to this Article.

## Supplementary Information

Supplementary Information

Reporting Summary

## Data Availability

The simulation data and code used in this study are available in the nonlinearPGG repository at https://github.com/MathOnco/nonlinearPGG.
