# Supplementary material for: Neighborhood size-effects shape growing population dynamics in evolutionary public goods games
Source: Commun Biol. 2019 Feb 5;2:53. doi: 10.1038/s42003-019-0299-4 (PMC6363775; doi:10.1038/s42003-019-0299-4)
Supplement: Supplementary file 1 — Supplementary Information [file 42003_2019_299_MOESM1_ESM.pdf]

Supplementary Figures

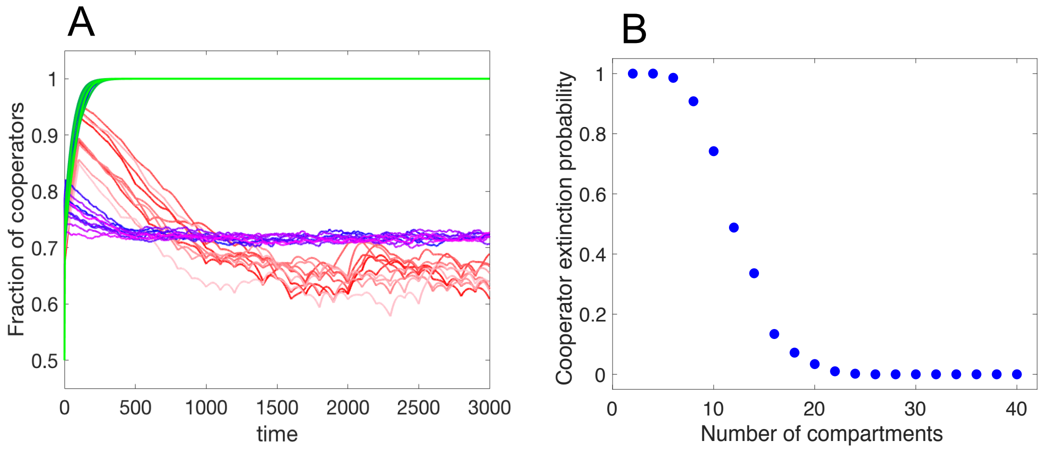

Supplementary Figure 1: Stochastic effects of the N-compartment model. A The impact of selection time $T_s = 10, 100, 1000$, blue, red and green, respectively, with ten trajectories given for each. Parameters used $N = 40,\ \alpha = 1,\ \sigma = 2,\ \beta = 5,\ \kappa = 0.5,\ \delta_C = \delta_D = 0.1,\ C_0 = D_0 = 50$. **B** $T_s = \infty$ (no redistribution after initialization). The producer extinction probability by number of compartments. Parameters used $\alpha = 1,\ \sigma = 3,\ \beta = 2,\ \kappa = 0.5,\ \delta_C = \delta_D = 0.1,\ C_0 = D_0 = 50$.

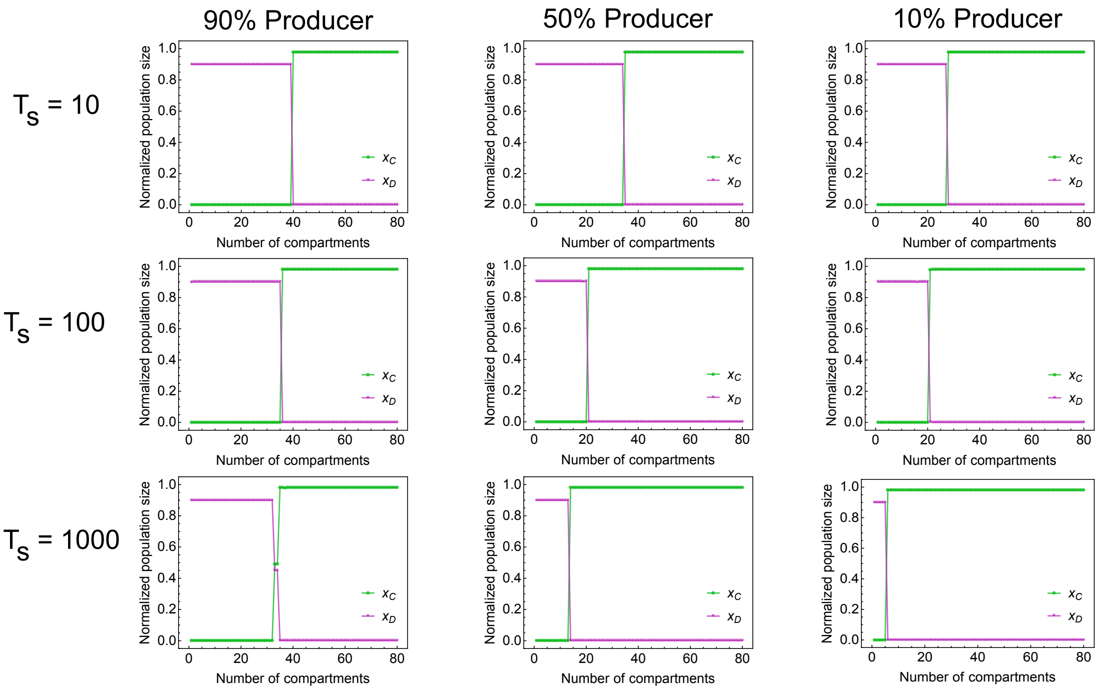

Supplementary Figure 2: Critical compartment number and stable state-switching in the N-compartment model. Parameters: $\beta = 2$, $\sigma = 3$, $\alpha = 1$/day, $\delta = 0.1$/day, $\kappa = 0.5$/day, $K = 1000$.

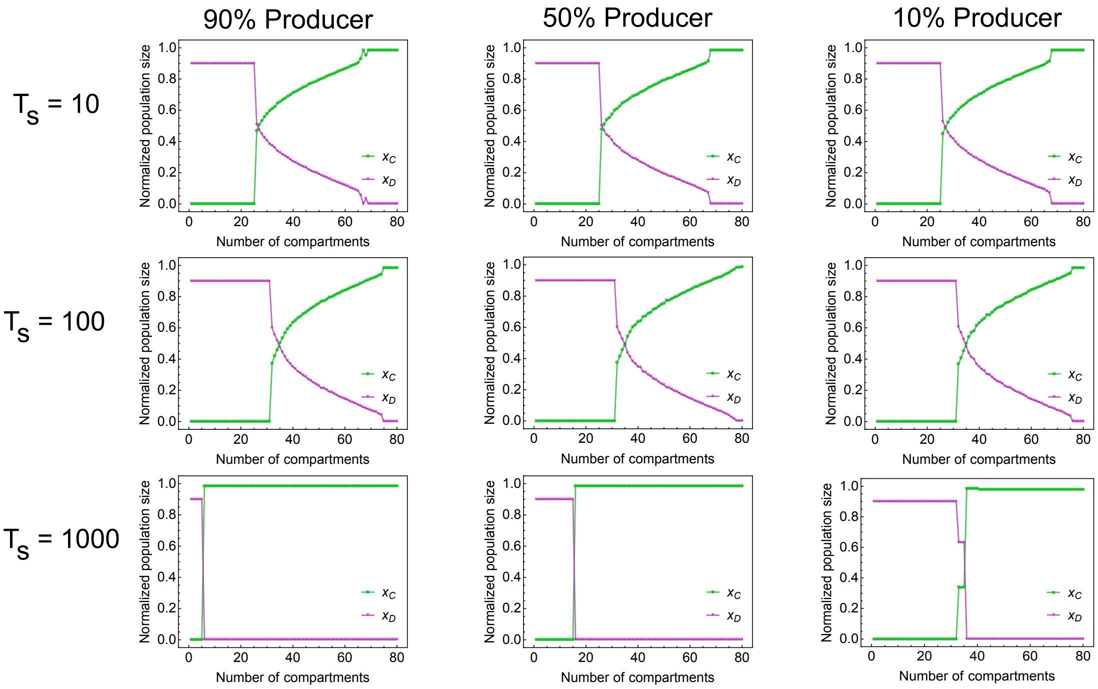

Supplementary Figure 3: Critical compartment number and coexistence in the N-compartment model. Parameters: $\beta = 5$, $\sigma = 2$, $\alpha = 1$/day, $\delta = 0.1$/day, $\kappa = 0.5$/day, $K = 1000$.

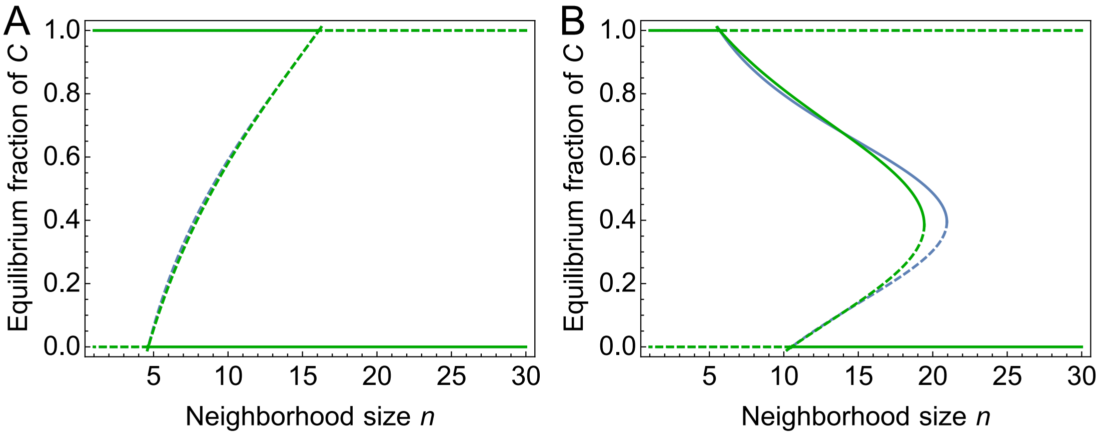

Supplementary Figure 4: Model deviations. To obtain the green curves we reversed the order of calculating expected neighborhood compositions for C and D. Blue curve is the bifurcation curve from the main text. **A** Deviation for small β with a transcritical bifurcation. **B** Deviation for large β with a transcritical bifurcation and saddle-node bifurcation. Parameters in **A**: $\beta = 2$, $\sigma = 3$, $\alpha = 1$/day, $\delta = 0.1$/day, $\kappa = 0.5$/day, $K = 1000$. Parameters in **B**: $\beta = 5$, $\sigma = 2$, $\alpha = 1$/day, $\delta = 0.1$/day, $\kappa = 0.5$/day, $K = 1000$.

Supplementary Methods

Contents

1 Individual-based birth and death model 3
- 1.1 Stochastic model and master equation 3
- 1.2 Passage to the deterministic case 4
- 1.3 Stochastic simulations 4
- 1.4 Neighborhood composition 5

2 Fallacy of averages 6

3 Co-evolution of cooperators and defectors in a public goods game 7
- 3.1 Equilibria points 8
- 3.2 Generalized model with K-selection competition matrix 8

4 Linear stability analysis 9
- 4.1 Boundary equilibria 9
- 4.2 Internal equilibria 9
- 4.3 Equal death rates - impossibility of closed orbits 12

5 Nonlinear model fit 12

6 Fluctuations in n: quasi-spatial model analysis 13
- 6.1 Critical compartment size 13

1 Individual-based birth and death model

1.1 Stochastic model and master equation

Let C be the number of cooperators and D the number of defectors. Then, the stochastic process which governs the population dynamics is given by,

$$C \xrightarrow{\lambda_C} C + C, \tag{S1a}$$

$$D \xrightarrow{\lambda_D} D + D, \tag{S1b}$$

$$C \xrightarrow{\delta_C} \emptyset, \tag{S1c}$$

$$D \xrightarrow{\delta_D} \emptyset, \tag{S1d}$$

where

$$\lambda_i = r_i(y)\left(1 - \frac{C+D}{K}\right). \tag{S2}$$

We note here that $r_i(y) = r_i(x_C/(x_C + x_D))$ is unchanged by considering populations and not concentrations. This can be seen by defining $x_i = i/K$, then

$$\frac{C}{C+D} = \frac{Kx_C}{Kx_C + Kx_D} = \frac{x_C}{x_C + x_D} = y.$$

The transition rates are then given by

$$T_i^+ = r_i(y)C\left(1 - \frac{C+D}{K}\right), \tag{S3a}$$

$$T_i^- = \delta_i i, \tag{S3b}$$

with $i = C, D$. We are now able to write down the master equation

$$\frac{\partial P}{\partial t} = T_{C-1}^+ P_{C-1} + T_{C+1}^- P_{C+1} + T_{D-1}^+ P_{D-1} + T_{D+1}^- P_{D+1} - (T_C^+ + T_C^- + T_D^+ + T_D^-)P. \tag{S4}$$

Unlike the deterministic model, this stochastic model has absorbing states at $C = 0$ or $D = 0$. Hence, states which are unstable in the ODE model are not unstable in the stochastic version.

1.2 Passage to the deterministic case

For $K \gg 1$, it is possible to connect the stochastic model to the deterministic model by making the transformation $n_C = C/K$ and $n_D = D/K$. The transition rates become

$$T_{n_i}^+ = r_i(y)Kn_i\left(1 - n_C - n_D\right), \tag{S5a}$$

$$T_{n_i}^- = \delta_i K n_i. \tag{S5b}$$

Subjecting the master equation (S4) to a system-size expansion expansion (with $1/K \ll 1$) [1], we arrive at a Fokker-Planck equation that describes the associated diffusion process [2]

$$\frac{\partial P}{\partial t} = -\frac{1}{K}\frac{\partial}{\partial n_i}\left[\left(T_{n_i}^+ - T_{n_i}^-\right)P\right] + \frac{1}{2K^2}\frac{\partial^2}{\partial n_i^2}\left[\left(T_{n_i}^+ + T_{n_i}^-\right)P\right]. \tag{S6}$$

To connect with the deterministic model we now assume $K \to \infty$ and so we keep only the first term. Multiplying both sides of Eq. (S6) by n_C and integrating over all possible values. The left-hand side is,

$$\int\int n_C \frac{\partial P}{\partial t}\, dn_C dn_D = \frac{d\langle n_C\rangle}{dt} = \frac{dx_C}{dt},$$

where we have defined $x_i = \langle n_i \rangle$. The right hand side is

$$-\int\int n_C \frac{\partial}{\partial n_C}[(T_{n_C}^+ - T_{n_C}^-)P]\, dn_C dn_D = -\left\{\int n_C[(T_{n_C}^+ - T_{n_C}^-)P]\Big|_{n_C=0}^{n_C=\infty} dn_D - \langle T_{n_C}^+ - T_{n_C}^-\rangle\right\} = \langle T_{n_C}^+ - T_{n_C}^-\rangle.$$

It is easy to show that the second term will go to zero since $\frac{\partial n_C}{\partial n_D} = 0$. A similar computation will result in an ODE for x_D, and so we obtain

$$\frac{dx_C}{dt} = \frac{1}{K}\langle T_{n_C}^+ - T_{n_C}^-\rangle = r_C(y)x_C(1 - x_C - x_D) - \delta_C x_C, \tag{S7a}$$

$$\frac{dx_D}{dt} = \frac{1}{K}\langle T_{n_D}^+ - T_{n_D}^-\rangle = r_D(y)x_D(1 - x_C - x_D) - \delta_D x_D, \tag{S7b}$$

which we recognize as a re-scaled version of Eq. (2).

1.3 Stochastic simulations

Now that we have established correspondence between the stochastic and deterministic models, we can investigate the impact of stochasticity in this system. We used the Gillespie algorithm to generate trajectories of the stochastic system.

Simulations were carried out using Julia 0.0.7 according to the following scheme:

(a) **Initialization:**

Set biological parameters (e.g. α, β, neighborhood size).

(b) **Dynamics loop:**

(1) Determine random composition of neighborhood seen by a cooperator (N_C) or a defector (N_D).

(2) Calculate transition rates.

(3) Determine time till next event (e.g. birth or death of either a cooperator or defector).

(4) Determine which event occurs (based on the transition rates).

(5) Update population sizes. Go back to (1).

(c) **Output:**

Dynamics of the total population $(t, C(t), D(t))$,

Final size $(C(t_{\text{final}}), D(t_{\text{final}}))$ and public good benefit observed.

We determine the neighborhood composition, we draw n individuals from the population using a hypergeometric distribution. In the main text, we took the expected neighborhood composition since it is impractical to compute this each iteration step using an ODE model which assumes continuous population sizes.

We see qualitatively similar results between the stochastic and deterministic models for large population sizes (Figures 1, 2). However, the stochastic model admits a nonzero probability of extinction of either population in all cases, which is not possible in the deterministic setting. In addition, a region of coexistence is observed when $\beta = 2,\ \sigma = 3$ which was not predicted in the deterministic model. This discrepancy can best be explained by the fact that the Gillespie algorithm was only run up to 100,000 events. It is likely in this region of parameter space that a long time might be needed to observe the extinction events predicted by the ODE model.

1.4 Neighborhood composition

To obtain a group of n individuals drawn from a total population of size $C + D$ with C individuals are cooperators, we use the hypergeometric distribution. The probability of selecting $C = k$ cooperators in the group is given by

$$P(C = k) = \frac{\binom{C}{k}\binom{D}{n-k}}{\binom{C+D}{n}}. \tag{S8}$$

In our particular scenario, we are more interested in the probability of selecting $n-1$ individuals to fill the remaining group and determining the size given that particular individual who is observing the neighborhood. The probability that we have k cooperators in a group given the individual observing is a cooperator is then given by

$$P(C = k|\text{one cooperator selected}) = \frac{\binom{C-1}{k-1}\binom{D}{n-k}}{\binom{C+D-1}{n-1}}, \tag{S9a}$$

$$P(C = k|\text{one defector selected}) = \frac{\binom{C}{k}\binom{D-1}{n-k-1}}{\binom{C+D-1}{n-1}}. \tag{S9b}$$

In the stochastic simulations, we draw from these to determine the N_C, N_D at each iteration. In the deterministic version we took the expected value of these distributions. To do that we consider Eq. (S8) by multiplying by k and averaging over all possibilities. We first make note of the following relationship

$$k\binom{C}{k} = \frac{C!}{(k-1)!(C-k)!} = \frac{C(C-1)!}{(k-1)!(C-k)!} = C\binom{C-1}{k-1}.$$

$$\begin{aligned}
\langle k \rangle &= \frac{1}{\binom{C+D}{n}} \sum_{k=1}^{n} k \binom{C}{k} \binom{D}{n-k} \\
&= \frac{C}{\binom{C+D}{n}} \sum_{k=1}^{n} \binom{C-1}{k-1} \binom{D}{n-k} \\
&= \frac{C}{\binom{C+D}{n}} \sum_{k=0}^{n-1} \binom{C-1}{k} \binom{D}{n-k-1} \\
&= \frac{C}{\binom{C+D}{n}} \binom{C+D-1}{n-1} \\
&= \frac{nC}{C+D}.
\end{aligned}$$

Using the form in Eq. (S9a) we see that a cooperator observes an expected number of cooperators in the neighborhood given by

$$\hat{N}_C = \underbrace{1}_{\text{individual cooperator}} + \underbrace{\frac{(n-1)(C-1)}{C+D-1}}_{\text{expected cooperators in } n-1 \text{ spots}}. \tag{S10}$$

A similar relationship using Eq. (S9b) is observed for a defector

$$\hat{N}_D = \frac{(n-1)C}{C+D-1}. \tag{S11}$$

The perceived expected proportion is then given by dividing by the neighborhood size,

$$\tilde{N}_C = \frac{1}{n}\left[1 + (n-1)\frac{(C-1)}{C+D-1}\right], \tag{S12a}$$

$$\tilde{N}_D = \frac{1}{n}(n-1)\frac{C}{C+D-1}. \tag{S12b}$$

For $C, D \gg 1$ we see that

$$\tilde{N}_D \approx \frac{1}{n}(n-1)\frac{C}{C+D}\left(1+\frac{1}{C+D}\right) = \frac{1}{n}(n-1)y\left(1+\frac{1}{C+D}\right) = N_D\left(1+\frac{1}{C+D}\right).$$

Hence, $\tilde{N}_D - N_D \approx N_D(C+D)^{-1}$. This implies that the deterministic version has a tendency to underestimate the proportion of cooperation in the neighborhood observed by a defector. In contrast, we see that

$$\tilde{N}_C \approx \frac{1}{n}\left[1+(n-1)\frac{(C-1)}{C+D}\left(1+\frac{1}{C+D}\right)\right] = \frac{1}{n}\left[1+(n-1)\left(y-\frac{1}{C+D}\right)\left(1+\frac{1}{C+D}\right)\right] = N_C - \frac{n-1}{n}\frac{1-y}{C+D}.$$

Hence, $\tilde{N}_C - N_C \approx -(1-1/n)(1-y)(C+D)^{-1}$. This implies that the deterministic version has a tendency to overestimate the proportion of cooperation in the neighborhood observed by a cooperator. For large population sizes, the expected neighborhood composition calculated through the hypergeometric distribution approaches that of the binomial distribution.

2 Fallacy of averages

In the Main Text, we supposed that the cells see their expected neighborhood, and then calculated growth rates that incorporated these expectation values. However, one may also consider the expected nonlinear public good payoff, that is one could calculate averages at a different point. Mathematically, this is equivalent to comparing

$E[f(x)]$ and $f[E(x)]$ for a nonliear function f. In general, these operations are not the same, however, we can show in this case that the qualitative results are unchanged. Suppose the populations and neighborhood sizes are large enough such that we can consider a continuous probability distribution (e.g. normal approximation to a binomial distribution). Let $E[x] = \mu$ and expand $f(x)$, about μ, where $x = \mu + h$ and $h \ll 1$. Taking a Taylor series and keeping terms only to second order, we obtain

$$f(x) = f(\mu + h) = f(\mu) + hf'(\mu) + \frac{1}{2}h^2 f''(\mu) + O(h^3). \tag{S13}$$

Taking the expectation of both sides and noting that $E(h) = E(x - \mu) = 0$ and $E(h^2) = E[(x - \mu)^2] = \text{var}(x)$, we obtain

$$E[f(x)] \approx f(\mu) + \frac{1}{2}f''(\mu)\text{var}(x). \tag{S14}$$

Assuming that the population and neighborhood size is sufficiently large, we suppose $N_D \sim \mathcal{N}[y(n-1), \sqrt{y(1-y)(n-1)}]$. We can quantify the effect of switching the order of operations in Eq. (4b) by analyzing the second term in Eq. (S14),

$$\frac{1}{2}\text{var}(x)\frac{\partial^2 r_D(N_D)}{\partial N_D^2}\bigg|_{N_D=E(N_D)} = \frac{\alpha_D(1+e^\sigma)\beta^2(n-1)(1-y)ye^{\frac{\beta y}{n}+\sigma+\beta y}\left(e^{\frac{\beta y}{n}+\sigma} - e^{\beta y}\right)}{2n^2\left(e^{\frac{\beta y}{n}+\sigma} + e^{\beta y}\right)^3}. \tag{S15}$$

A similar result is obtained for N_C. By abuse of notation, let the main text $r_i = r_i(E[N_i])$ and define $\tilde{r}_i = E[r_i(N_i)]$. Then $\tilde{r}_i \approx r_i + \text{var}(N_i)r_i''(E[N_i])/2$. We now summarize the impact by replacing r_i in the main text with $\tilde{r}_i$:

- In the absence of producers or cooperators, $\text{var}(N_i) \to 0$ and so $\tilde{r}_i \approx r_i$.
- *The boundary equilibria stability will be unchanged.* This can be noted by observing Eq. (S23)-(S24). The eigenvalues are dependent on $r_i(0)$ or $r_i(1)$. Again, we would expect $\tilde{r}_i \approx r_i$. Additionally, the time will also be unchanged, however the eigenvectors are modified, which will shift the directions trajectories take to approach the boundary equilibria.
- *The location of coexistence points will shift.* By noting the coexistence condition Eq. (S19), we can see that

$$\frac{\delta_C}{\delta_D} = \frac{r_C + \text{var}(N_C)r_C''(E[N_C])/2}{r_D + \text{var}(N_D)r_D''(E[N_D])/2}. \tag{S16}$$

- *The stability of coexistence points will shift.* By looking at Eq. (S34), we see that the stability condition is dependent on the values of $\tilde{r}_i$ at $y = y^*$.

These results are consistent with those observed in figure 4.

3 Co-evolution of cooperators and defectors in a public goods game

To begin, we rescale the populations $x_i \to Kx_i$ and so Eq (2) becomes

$$\frac{dx_C}{dt} = r_C\, x_C(1 - x_C - x_D) - \delta_C\, x_C, \tag{S17a}$$

$$\frac{dx_D}{dt} = r_D\, x_D(1 - x_C - x_D) - \delta_D\, x_D. \tag{S17b}$$

We map the dynamical system from producer/defector population to producer frequency and total population. We define $Y = x_1 + x_2$ and $y = x_1/Y$ and Eq. (S17) becomes

$$\frac{dy}{dt} = y(1-y)[(1-Y)(r_C - r_D) + \delta_D - \delta_C], \tag{S18a}$$

$$\frac{dY}{dt} = -Y\{\delta_C y + \delta_D(1-y) - (1-Y)[y r_C + (1-y) r_D]\}. \tag{S18b}$$

Based on the form given in Eq. (4), we analyze public good functions of the form $r_C = \alpha_C F_C - \kappa$ and $r_D = \alpha_D F_D$, where we set $F_i(0) = 1$, but we note a similar analysis can be done with $r_C = \alpha_C + G_C - \kappa$, $r_D = \alpha_D + G_D$, where $G_i(0) = 0$. The correspondence between the two forms is given by the relation $G_i = \alpha_i(F_i - 1)$. We call α_i the public-good independent (intrinsic) growth rate.

3.1 Equilibria points

The system governed by Eq. (S18) admits a minimum of two fixed points: the boundary equilibria. The (y^*, Y^*) are given by

- *Defectors win*: $\left(0, 1 - \frac{\delta_D}{r_D(0)}\right)$.
- *Cooperators win*: $\left(1, 1 - \frac{\delta_C}{r_C(1)}\right)$.

Internal equilibria can exist provided that two conditions are met. First, both death rates are either both zero or both nonzero. If $\delta_C = \delta_D = 0$, then coexistence is achieved with $Y^* = 1$ and y is undetermined (a line of non-isolated fixed points). If we suppose that δ_C, δ_D are both positive, then we arrive at the *coexistence condition*

$$\frac{r_C(y^*)}{r_D(y^*)} = \frac{\delta_C}{\delta_D}, \tag{S19}$$

where $Y^* = 1 - \frac{\delta_i}{r_i(y^*)}$. Finally we note that all physical trajectories ($y \in [0,1]$ and $Y \geq 0$) eventually enter the unit box $[0,1] \times [0,1]$. To see this we only need to note that if $Y > 1$, then both terms in Eq. (S18b) are positive and hence $\dot{Y} < 0$.

3.2 Generalized model with K-selection competition matrix

Instead of assuming an identical carrying capacities for all subpopulations, one can introduce a more general version of the rescaled model (S17) [3, 4], written as

$$\frac{dx_C}{dt} = r_C\, x_C(1 - a x_C - b x_D) - \delta_C\, x_C, \tag{S20a}$$

$$\frac{dx_D}{dt} = r_D\, x_D(1 - c x_C - d x_D) - \delta_D\, x_D. \tag{S20b}$$

Defining y and Y as before, we arrive at

$$\frac{dy}{dt} = y(1-y)\{r_C[1 - Y(A\vec{u})_1] - r_D[1 - Y(A\vec{u})_2] + \delta_D - \delta_C\}, \tag{S21a}$$

$$\frac{dY}{dt} = -Y\{\delta_C y + (1-y)\delta_D - r_C y[1 - Y(A\vec{u})_1] - r_D(1-y)[1 - Y(A\vec{u})_2]\}, \tag{S21b}$$

where we defined A the competition matrix and $\vec{u} = (y\ 1-y)^T$ the population frequency vector (e.g. $(A\vec{u})_i = A_{i1}y + A_{i2}(1-y)$). Note that when $a = b = c = d = 1$, $(A\vec{u})_i = 1$ and the model reduces to Eq. (S18). The

boundary equilibria remain, but the coexistence points are now given by a modified version of Eq. (S19),

$$\frac{\delta_C}{r_C} - \frac{\delta_D}{r_D} = \frac{(c-a)y + (d-b)(1-y)}{Y}, \tag{S22}$$

where we see it reduces if $a = c$ and $b = d$ (of which our original model is a special case). We analyze the stability of the boundary points:

- *Defectors win*: $\left(0, 1 - \frac{1}{d}\frac{\delta_D}{r_D(0)}\right)$.
- *Cooperators win*: $\left(1, 1 - \frac{1}{a}\frac{\delta_C}{r_C(1)}\right)$.

The all-D state is stable if $(2d-1)(\delta_D - r_D(0)) < 0$ and $\delta_D(1-d) - \delta_C + r_C(0)(1-b) + b\,\delta_D r_C(0)/r_D(0) - r_D(0)(1-d) < 0$. Assuming that $r_D(0) > \delta_D$ (as before), we see that $d > 1/2$ is required for stability.

In a similar way, the all-C state is stable if $(2a-1)(\delta_C - r_C(1)) < 0$ and $\delta_C(1-a) - \delta_D - r_C(1)(1-a) + r_D(1)(1-c) + c\,\delta_C r_D(1)/r_C(1) < 0$. Assuming that $r_C(1) > \delta_C$ (as before), we see that $a > 1/2$ is required for stability.

4 Linear stability analysis

4.1 Boundary equilibria

We analyzed the linear stability of the defector-only state first and obtained the Jacobian

$$J_{\text{all D}} = \begin{bmatrix} \frac{\delta_D r_C(0)}{r_D(0)} - \delta_C & 0 \\ -\frac{(\delta_D - r_D(0))\left\{\delta_D\left[r_C(0) + r_D'(0)\right] - \delta_C r_D(0)\right\}}{r_D(0)^2} & \delta_D - r_D(0) \end{bmatrix}. \tag{S23}$$

The eigenvalues of this system are $\lambda_1 = \delta_D - r_D(0)$, $\lambda_2 = \frac{\delta_D r_C(0)}{r_D(0)} - \delta_C$. This state is stable provided both eigenvalues are negative, which leads to two conditions: (1) $r_D(0) > \delta_D$, and (2) $r_D(0)/\delta_D > r_C(0)/\delta_C$. The first condition simply states the reasonable assertion that the intrinsic growth rate of defectors must exceed its respective death rate. The second condition states that the relative growth rate (the ratio of growth rate to death rate) of defectors exceeds that of producers.

The stability of the producer-only state is analyzed via the Jacobian

$$J_{\text{all C}} = \begin{bmatrix} \frac{\delta_C r_D(1)}{r_C(1)} - \delta_D & 0 \\ \frac{(\delta_C - r_C(1))\left(\delta_C\left(r_D(1) - r_C'(1)\right) - \delta_D r_C(1)\right)}{r_C(1)^2} & \delta_C - r_C(1) \end{bmatrix}. \tag{S24}$$

The two eigenvalues are $\lambda_1 = \delta_C - r_C(1)$, $\lambda_2 = \frac{\delta_C r_D(1)}{r_C(1)} - \delta_D$. This state is stable provided that both eigenvalues are negative, which leads to two conditions: (1) $r_C(1) > \delta_C$, and (2) $r_C(1)/\delta_C > r_D(1)/\delta_D$. The first condition simply states the reasonable assertion that the intrinsic growth rate of producers must exceed its respective death rate. The second condition states that the relative growth rate of producers exceeds that of defectors. Note the similarity in stability criterion between the two states.

4.2 Internal equilibria

What if both states are unstable? To answer this, we consider the function $\Gamma(y) = r_C(y) - \frac{\delta_C}{\delta_D} r_D(y)$. Then we see that the "all C" state is unstable provided that $\Gamma(1) < 0$ and the "all D" state is unstable provided that $\Gamma(0) > 0$. By the intermediate value theorem, there exists a $y^* \in (0,1)$ such that $\Gamma(y^*) = 0$, which we recognize from Eq. (S19) as the condition for a coexistence point. This shows the perhaps unsurprising fact that unstable boundary points in this system necessitate the existence of internal equilibria. It does not prove that the coexistence point is *stable*. Indeed, if there are stable closed orbits, a stable internal fixed point is not required. However, if the impossibility

of closed orbits can be deduced, we can conclude that at least one internal fixed point must be stable. We will show later the impossibility of closed orbits in the case of equal death rates (e.g. $\delta_C = \delta_D = \delta$).

It is useful to try to determine the maximum possible number of internal equilibria that can exist given a set of frequency-dependent growth rate function functions $r_C(y), r_D(y)$. We are interested then in the number of possible zeros of Eq. (S19), the y such that $\Gamma(y) = 0$. It is sometimes easier to locate extrema rather than zeros of a function. The correspondence between the number of zeros and extrema can be given by the following lemma:

Lemma. *Let P be the set of all coexistence points and E be the set of all extrema of the function Γ. The maximum number of coexistence points is given by $|P| = |E| + 1$.*

Proof. It is clear that a horizontal line can cross a function near an extrema at most two times. Ordering the extrema $E_1 < E_2 < \cdots < E_n$, we note that the interior extrema intersection points are counted twice. Hence, the number of possible coexistence points is given by $|P| = 2|E| - (|E| - 1) = |E| + 1$ which proves the claim. □

With this lemma, the problem of determining the possible number of coexistence points is equivalent to determining the possible number of extremum of Γ. At this point we must specify a form of the public good function. We then investigate the sensitivity of the number of coexistence points to different public good functions. Extrema are located at the zeros of the function

$$g(y) := F_C'(y) - \gamma F_D'(y), \tag{S25}$$

where we have defined $\gamma = \frac{\delta_C}{\delta_D}\frac{\alpha_D}{\alpha_C}$. We analyzed the existence of internal equilibria for four types of functions with the following properties

- The public good always benefits the population ($F_i(0) > 1$).
- More public good never hurts ($F_i'(y) > 0$ for all y).
- The good (unless linear) should eventually saturate ($F_i(y) \to F_\infty$ as $y \to \infty$).

These three properties describe many types of public goods. A notable exception was studied in the explanation of the Warburg effect, where the products of glycolysis are the public good used by cancer cells and at high quantities are actually harmful to the population [5].

First, consider the "almost identical" public good function $r_C/r_D = A = const$. Eq. (S25) becomes

$$g(y) = F_C'(y) - \gamma F_D'(y) = F_D'(y)[A - \gamma]. \tag{S26}$$

Since $F_i'(y) \geq 0$, we conclude that $g(y)$ is bounded away from 0 and hence there are no zeroes of g. This implies no extremum and so by the lemma there can be at most one internal equilibria. A similar result holds for the "always better" public good function. It is clear that if $F_C'(y) > \gamma F_D'(y)$ or $F_C'(y) < \gamma F_D'(y)$, then g will have no zeros and we can conclude at most one internal equilibrium point is possible. These general results show immediately that the linear public good can never have more than one coexistence point and hence saddle-node bifurcations and other interesting phase diagrams are not possible. Note, these results are independent of the listed properties.

A general class of models which satisfies the three properties listed above are sigmoidal functions. We consider two general forms: the Fermi-like function considered in the main text, and the Hill function.

The Hill function

$$F_i(y) = \frac{b_i y_i^{h_i}}{d_i + y^{h_i}} \tag{S27}$$

where the coefficients are all assumed to be nonnegative and $h_i \in \mathbb{Q}$ is the Hill coefficient. Plugging this into Eq. (S25), and setting to 0 leads to

$$\begin{aligned} g_{\text{Hill}}(y) = &- \gamma b_2 d_2 h_2 y^{2h_1+h_2} + b_1 d_1 h_1 y^{h_1+2h_2} \\ &+ 2d_1 d_2 (b_1 h_1 - \gamma b_2 h_2) y^{h_1+h_2} \\ &+ b_1 d_1 d_2^2 h_1 y^{h_1} - \gamma b_2 d_1^2 d_2 h_2 y^{h_2} \end{aligned} \tag{S28}$$

which we set to zero. There are three cases $h_1 > h_2$, $h_1 < h_2$ and $h_1 = h_2$. Let us investigate the case $h_1 > h_2$ first. Using Descartes' rule of signs (DRoC), we obtain $(-, +, \mathrm{sgn}(b_1h_1 - \gamma b_2h_2), +, -)$. Thus, DRoC shows that we can have at most four positive zeros if the middle term is positive and two if it is negative. The case $h_1 < h_2$ is analogous and leads to the same conclusion (with the condition on the middle term being reversed). The case $h := h_1 = h_2$ is special since this combines many of the terms in Eq. (S28): we require

$$g_{\mathrm{Hill}}(y) = hy^h[(b_1d_1 - \gamma b_2d_2)y^{2h} + 2d_1d_2(b_1 - \gamma b_2)y^h + d_1d_2(b_1d_2 - \gamma b_2d_1)] = 0. \tag{S29}$$

We can thus show that one cannot have alternating signs–hence we can only have at most *one* positive zero. Suppose the signs would alternate. Then we see that

$$b_1d_1 > \gamma b_2d_2 \tag{S30a}$$
$$b_1 < \gamma b_2 \tag{S30b}$$
$$b_1d_2 > \gamma b_2d_1 \tag{S30c}$$

Eq. (S30b) plugged into Eq. (S30a) implies $d_1 > d_2$, while Eq. (S30b) plugged into Eq. (S30c) implies that $d_2 > d_1$, a contradiction. Hence, signs cannot alternate. To briefly summarize the results of the Hill function,

- If $h_1 \neq h_2$, there are at most **five** internal equilibria.
- If $h_1 = h_2$, there are at most **two** internal equilibria.

Consider the Fermi benefit function $F_i(y) = \frac{1+\exp(\sigma)}{1+\exp[\sigma-\beta N_i(y)]}$ from Eq. (4) where $N_i(y)$ is the expected group composition $N_D(y) = \frac{(n-1)y}{n}$ and $N_C(y) = N_D(y) + \frac{1}{n}$. Plugging this into Eq. (S25) gives

$$g_{\mathrm{Fermi}}(y) = \left(e^{\beta N_D(y)} + e^{\sigma}\right)^2 - \gamma e^{-\frac{\beta}{n}}\left(e^{\beta N_C(y)} + e^{\sigma}\right)^2 = 0. \tag{S31}$$

Simplification leads to

$$e^{\beta N_D(y)} - e^{\sigma}\frac{1 - e^{-\frac{\beta}{2n}}\sqrt{\gamma}}{1 - e^{\frac{\beta}{2n}}\sqrt{\gamma}} = 0. \tag{S32}$$

As before, this has a positive zero if the signs alternate. It is easy to show that the second term is always negative and so there exists a positive zero of this function. Hence, the Fermi function can have at most **two** internal equilibria. This should not be too surprising if we note the transformation of the Hill function via $y = e^x$. The Hill function then transforms to

$$\tilde{F}_i(x) = \frac{b_i}{1 + d_ie^{-h_ix}}. \tag{S33}$$

Letting $b_i = 1 + e^{\sigma}$, $d_D = e^{\sigma}$, $d_C = e^{\sigma+\frac{\beta}{n}}$ and $h_i = \beta\left(\frac{n-1}{n}\right)$ is the Fermi function used in the main text. Noting that a monotonic transformation conserves the location of extrema, we immediately see that the properties of the Fermi function actually follow as a corollary from the properties of the general Hill function considered. This is because h_i are equal and when $h_1 = h_2$ we showed that there can be at most two internal equilibria.

The general stability of the internal equilibria is much more complex. The Jacobian of an internal equilibria (y^*, Y^*) is given by

$$J_{\mathrm{coexist}} = \begin{bmatrix} y(1-y)(1-Y)\,(r'_C - r'_D) & y(1-y)(r_D - r_C) \\ Y(1-Y)\,[yr'_C + (1-y)r'_D] & -Y[yr_C + (1-y)r_D] \end{bmatrix} \tag{S34}$$

In this case, it is easier to look at the determinant Δ and trace τ. The values are

$$\Delta = y^*Y^*(1-y^*)(1-Y^*)(r_Cr'_D - r_Dr'_C), \tag{S35}$$
$$\tau = y^*(1-y^*)(1-Y^*)(r'_C - r'_D) - Y^*[y^*r_C + (1-y^*)r_D]. \tag{S36}$$

A necessary condition for linear stability of the internal equilibria is that $\Delta > 0$, which is true if $r_C r_D' > r_D r_C'$, or using Eq. (S19) and (S25), $g(y^*) < 0$.

Let $\delta = \delta_1 = \delta_2$. The coexistence points occur at $r_C(y^*) = r_D(y^*)$, with $Y^* = 1 - \frac{\delta}{r_C(y^*)}$. We note here that for physical solutions we require $Y^* > 0$ which implies that $r_C(y^*) > \delta$. The Jacobian is

$$J = \begin{bmatrix} 0 & 0 \\ Y^*(1-Y^*)r_C'(y^*) & (1-2Y^*)r_C(y^*) - \delta \end{bmatrix} \tag{S37}$$

The eigenvalues are $\lambda = 0, (1-2Y^*)r_C(y) - \delta$. Based on the previous note of Y^* it is clear that the second eigenvalue is always negative and so all coexistence points are at least conditionally stable (there exists a trajectory which approaches the point). However, since one eigenvalue is 0, the nonlinear terms are relevant.

We want to find conditions that ensure stability of the coexistence points. Defining $u = y - y^*$, $v = Y - Y^*$ and inserting these into Eq. (S18) we obtain

$$\dot{u} = (u + y^*)(1 - y^* - u)\left[\frac{\delta}{r_C(y^*)} - v\right][r_C(y^* + u) - r_D(y^* + u)] \tag{S38}$$

If we are close to the equilibrium $(u, v \ll 1)$, we can replace $v = Cu$ where C is related to the entries in the Jacobian. Plugging this into the above and expanding Γ_i in small u we obtain

$$\dot{u} = (u + y^*)(1 - y^* - u)\left[\frac{\delta}{r_C(y^*)} - Cu\right]\left[\frac{u^k}{k!}r_C^{(k)}(y^*) - \frac{u^l}{l!}r_D^{(l)}(y^*)\right] \tag{S39}$$

where $r_C^{(k)}, r_D^{(l)}$ are the first nonzero terms in the expansion. Neglecting higher order terms we obtain

$$\dot{u} = \frac{\delta y^*(1-y^*)}{r_C(y^*)}\left[\frac{u^k}{k!}r_C^{(k)}(y^*) - \frac{u^l}{l!}r_D^{(l)}(y^*)\right]. \tag{S40}$$

If $k > l$ then the state is stable if $r_D^{(l)}(y^*) > 0$, and if $k < l$ then it is stable if $r_C^{(k)}(y^*) < 0$. If $k = l$ then the state is stable if $r_C^{(k)}(y^*) - r_D^{(k)}(y^*) < 0$.

4.3 Equal death rates - impossibility of closed orbits

By index theory, we require at least one non-saddle fixed point to be in the interior of a closed orbit. Only interior equilibria can satisfy this requirement (the closed orbit cannot leave the unit box in phase space). Furthermore, if the closed orbit surrounds more than one interior equilibria, it must be an odd number $2k+1$ such that there are $k+1$ nodes and k saddles, with $k \geq 0$. Consider the special case when $\delta_C = \delta_D = \delta$, then Eq. (S18) reduces to

$$\frac{dy}{dt} = y(1-y)(1-Y)(r_C - r_D), \tag{S41a}$$

$$\frac{dY}{dt} = -Y\{\delta - (1-Y)[yr_C + (1-y)r_D]\}. \tag{S41b}$$

The coexistence point condition reduces to $r_C(y^*) = r_D(y^*)$. It is clear that $\dot{y} = 0$ and $\dot{Y} = -Y[\delta - (1-Y)r_C(y^*)]$ on the line $y = y^*$. If $Y < Y^*$, then $\dot{Y} > 0$, while if $Y > Y^*$, $\dot{Y} < 0$. It is impossible for a closed orbit to not transverse the curve Φ. However, the line is also invariant (a trajectory cannot leave once it is on it). Hence closed orbits are not possible, since no trajectory can cross through the line $y = y^*$.

5 Nonlinear model fit

We used equation (S18a) with the assumption $Y \ll K$ ($(1-Y) \to 1$ in the rescaled dynamical system) to perform a nonlinear model fit in Wolfram Mathematica 11.2 (*NonlinearModelFit* and *ParametricNDSolve*) to obtain maximum

likelihood estimates of β and σ. We set $\alpha = 1/\text{day}$, as this would only alter the overall time scale and not the measured growth rate differences. We also set $\kappa = 0.25/\text{day}$, as estimated from Fig. 1 in [6], where under highest growth rate concentration the median difference between non-producer growth rate and producer growth rate amounted to 25% of the intrinsic growth rate. This fitting procedure was applied for every value of FBS concentration in the *in vitro* growth medium, for every integer value of n between 4 and 40, to result in 'distributions' of values of β and σ. These values of β and σ are reported in Figure 3, panels B and C.

6 Fluctuations in n: quasi-spatial model analysis

To simulate the effects of N-compartments subject to periodic mixing, we considered the $2N$-coupled system of ODEs given by

$$\frac{dx_{C,i}}{dt} = r_{C,i}x_{C,i}\left[1 - \frac{x_C + x_D}{K}\right] - \delta_C x_{C,i}, \tag{S42a}$$

$$\frac{dx_{D,i}}{dt} = r_{D,i}x_{D,i}\left[1 - \frac{x_C + x_D}{K}\right] - \delta_D x_{D,i}, \tag{S42b}$$

where $i = 1, \ldots, N$ and it is understood that $x_k = \sum_{i=1}^{N} x_{k,i}$. Each compartments dynamics are governed by the local interplay of producers and defectors within the compartment. The coupling between compartments is through the carrying capacity K.

Simulations were carried out in MATLAB according to the following scheme:

(a) **Initialization:**

Set biological parameters (e.g. α, β, initial population size, number of compartments).

Distribute individuals among the compartments according to multinomial distribution.

(b) **Dynamics loop:**

(1) Integrate Eq. (S42) from $[t_0, t_0 + T_s]$ for fixed T_s.

(2) If exit criterion is met, break out of the loop.

(3) Redistribute cells according to the multinomial distribution. Go back to (1).

(c) **Output:**

Dynamics of the total population $(t, x_C(t), x_D(t))$,

Final size $(x_C(t_{\text{final}}), x_D(t_{\text{final}}))$.

The exit criterion was met when $x_C x_D = 0$ for some t or if equilibration was reached. This was done by looking back a given amount of time steps and comparing the mean to the current value. After a set number of times they were within a certain tolerance, the loop would end. To solve the ODE, MATLAB's ode45 built-in solver was used.

Aside from the compartments, all biological parameters are assumed fixed in the entire system (e.g. there is no compartment-dependent β). However, a new parameter - the selection time T_s is necessary. We investigated the impact of T_s for fixed compartment size. An interesting effect is observed in Fig. 1. For $T_s \to \infty$, mixing never takes place and in this scenario, the defectors go extinct. For finite T_s, one observes coexistence. However, as $T_s \to 0$ (time scales of mixing and selection are the same), the population appears to be moving towards fixation.

6.1 Critical compartment size

It is clear that N is inversely related to the neighborhood size. Therefore, it is not surprising that there should exist some critical number of compartments at which the producers should be favored. To see this, note that $N = 1$ is the original case where the neighborhood is the whole population. The defectors are the winners as this is the classic tragedy of the commons problem. On the other side, if $N \to \infty$, each compartment will have at most one

individual and hence the neighborhood size is effectively 1. In this case, the producers should win. We then expect there to exist some N_{crit} such that for $N > N_{\text{crit}}$, producers are favored.

To approximate this, we use a mean-field approach where we replace the population size in each compartment by its expected value. We analyze the stability of the all producer population $y_i = 1$ for all i. We require the population size to be $Y = 1 - \frac{\delta_C}{r_{C,i}(1)}$. We replace Y_i by its expected value of Y/N. This collapses the N-compartment model to the original model and we recall the eigenvalues of the "all C" state $\lambda_1 = \delta_C - r_C(1)$ and $\lambda_2 = \frac{\delta_C r_D(1)}{r_C(1)} - \delta_D$. Assuming the birth rate exceeds the death rate, the first is satisfied. The critical curve is then determined by $\lambda_2 = 0$. Solving this for the critical neighborhood size n_{crit}, we obtain

$$\frac{1}{n_{\text{crit}}} = 1 - \frac{1}{\beta}\left[\sigma - \ln\left(\frac{\delta_C \alpha_D}{\delta_D \alpha_C}\frac{1-f}{f}\right)\right], \tag{S43}$$

with

$$f = \frac{1}{1 + e^{\sigma - \beta}} - \frac{\kappa}{\alpha_C(1 + e^{\sigma})}. \tag{S44}$$

Now, the neighborhood size is the population size in the compartment and so its average will be $\langle n \rangle = Y/N$. Plugging this into Eq. (S43) yields (after scaling back carrying capacity)

$$N_{\text{crit}} = K\left(1 - \frac{\delta_C}{r_C(1)}\right)\left\{1 - \frac{1}{\beta}\left[\sigma - \ln\left(\frac{\delta_C \alpha_D}{\delta_D \alpha_C}\frac{1-f}{f}\right)\right]\right\}, \tag{S45}$$

with

$$r_C(1) = \alpha_C \frac{1 + e^{\sigma}}{1 + e^{\sigma - \beta}} - \kappa. \tag{S46}$$

Supplementary References

[1] van Kampen NG. Stochastic Processes in Physics and Chemistry. 2nd ed. Amsterdam: Elsevier; 1997.

[2] Gardiner CW. Handbook of Stochastic Methods. 3rd ed. Springer, NY; 2004.

[3] McKane AJ, Newman TJ. Stochastic models in population biology and their deterministic analogs. Physical Review E. 2004;70:19.

[4] Huang W, Hauert C, Traulsen A. Stochastic game dynamics under demographic fluctuations. Proceedings of the National Academy of Sciences. 2015;112:9064–9069.

[5] Archetti M. Evolutionary dynamics of the Warburg effect: glycolysis as a collective action problem among cancer cells. Journal of theoretical biology. 2014;341:1–8.

[6] Archetti M, Ferraro DA, Christofori G. Heterogeneity for IGF-II production maintained by public goods dynamics in neuroendocrine pancreatic cancer. Proceedings of the National Academy of Sciences USA. 2015;112:1833–1838.
